# The novel Nrf2 activator CDDO‐EA attenuates cerebral ischemic injury by promoting microglia/macrophage polarization toward M2 phenotype in mice

**DOI:** 10.1111/cns.13496

**Published:** 2020-12-06

**Authors:** Xia Lei, Hanxia Li, Min Li, Qiwei Dong, Huayang Zhao, Zongyong Zhang, Baoliang Sun, Leilei Mao

**Affiliations:** ^1^ Department of Neurology Second Affiliated Hospital Shandong First Medical University & Shandong Academy of Medical Sciences Taian China; ^2^ Key Laboratory of Cerebral Microcirculation in Universities of Shandong Shandong First Medical University & Shandong Academy of Medical Sciences Taian China; ^3^ Department of Neurology Cangzhou People’s Hospital Cangzhou China

**Keywords:** CDDO‐EA, cerebral ischemia, HO‐1, microglia/macrophage, Nrf2

## Abstract

The aim of present study was to explore whether 2‐cyano‐3, 12‐dioxooleana‐1, 9‐dien‐28‐oic acid (CDDO)‐ethylamide (CDDO‐EA) attenuates cerebral ischemic injury and its possible mechanisms using a middle cerebral artery occlusion (MCAO) model in C57BL/6 mice. Our results showed that intraperitoneal injection (i.p.) of CDDO‐EA (2 and 4 mg/kg) augmented NFE2‐related factor 2 (Nrf2) and heme oxygenase‐1 (HO‐1) expression in ischemic cortex after MCAO. Moreover, CDDO‐EA (2 mg/kg, i.p.) significantly enhanced Nrf2 nuclear accumulation, associated with increased cytosolic HO‐1 expression, reduced neurological deficit and infarct volume as well as neural apoptosis, and shifted polarization of microglia/macrophages toward an antiinflammatory M2 phenotype in ischemic cortex after MCAO. Using an in vitro model, we confirmed that CDDO‐EA (100 μg/mL) increased HO‐1 expression and primed microglial polarization toward M2 phenotype under inflammatory stimulation in BV2 microglial cells. These findings suggest that a novel Nrf2 activator CDDO‐EA confers neuroprotection against ischemic injury.

## 1 INTRODUCTION

Cerebral injury caused by vascular obstruction can lead to ischemic stroke, which is a leading cause of disability in human and a high cost burden to society.^1^ Rapid restoration of blood supply through surgical treatment and intravenous thrombolysis is an effective treatment, although this also can lead to cerebral ischemia‐reperfusion injury.^2^ The pathophysiological mechanism of cerebral ischemia‐reperfusion injury is complex, involving cell apoptosis, oxidative stress injury, and inflammatory response.^3^, ^4^ When ischemic stroke occurs, local microglia/macrophages are rapidly activated, mobilize to the injury site, and initiate the release of effectors and recruitment of peripheral inflammatory cells.^3^, ^4^ Microglia/macrophages have high plasticity that can assume diametrically opposed functional phenotypes when responding to micro‐environmental triggers. One phenotype is the “classically activated” M1 that release destructive proinflammatory mediators. The polar extreme phenotype is “alternatively activated” antiinflammatory M2 phenotype that has been associated with neuroprotective effects. Recent studies suggest that modulation of microglia/macrophage polarization toward M2 phenotype may be harnessed as an important treatment strategy for brain repair in ischemic stroke.^5^, ^6^

**FIGURE 1:**
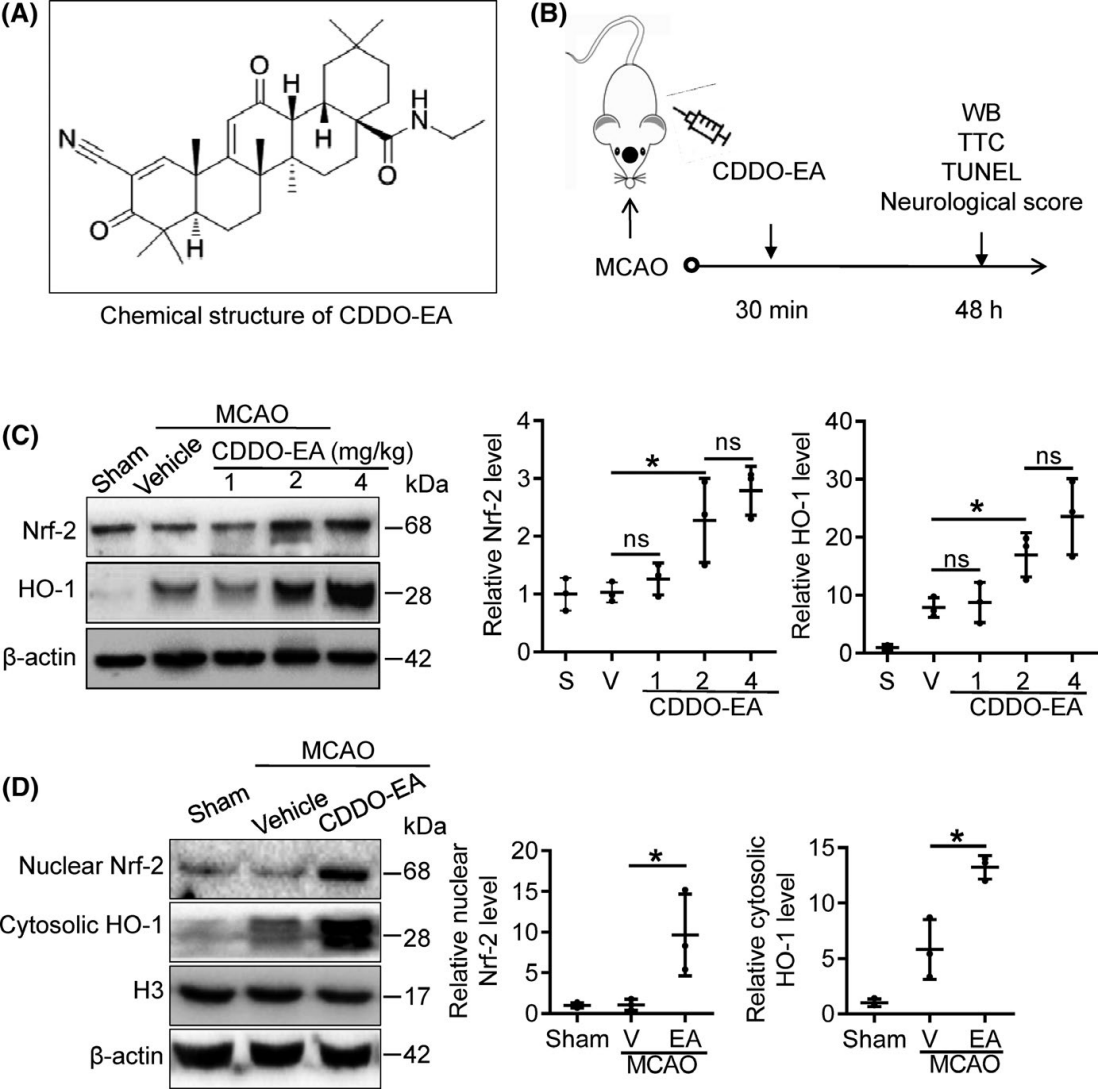
CDDO‐EA increases Nrf2 and HO‐1 expression in cortex after transient MCAO. A, Chemical structure of CDDO‐EA. B, Scheme for the experimental design. C, Nrf2 and HO‐1 protein expression in the ischemic cortex was detected by Western blot 48 h after MCAO. Mice were injected with CDDO‐EA (0, 1, 2, and 4 mg/kg, i.p.) 30 min after MCAO surgery. Cortexes were collected at 48 h after MCAO, and homogenates were blotted with anti‐Nrf2, anti‐HO‐1, and anti‐β‐actin. Quantification of optical density was normalized to sham controls. D, Mice were injected with CDDO‐EA (2 mg/kg, i.p.) followed by MCAO. Ischemic cortexes were collected at 48 h after MCAO and subjected to subcellular fractionation. Nuclear Nrf2 and cytosolic HO‐1 were detected by Western blot, and Histone H3 and β‐actin were used as loading controls for nuclear and total protein, respectively. Data are mean ± SD. (ns denotes not significant, ^*^ *P* < 0.05, n = 3)

Recent research indicates that activation of the redox transcription factor NFE2 related factor 2 (Nrf2) contributes to the antiinflammatory nature of the M2 microglia/macrophage phenotype.^7^, ^8^ Activated Nrf2 translocates to the nucleus and binds to the antioxidative response element (ARE) in cells exposed to oxidative stress, which triggers the transcription of antioxidant and antiinflammatory genes.^9^ Nrf2 and its downstream transcriptional target heme oxygenases‐1 (HO‐1) have neuroprotective effects against some models of ischemic injury in the central nervous system.^10^, ^11^, ^12^, ^13^ 2‐Cyano‐3,12‐Dioxooleana‐1,9‐Dien‐28‐Oic acid (CDDO) and its analogues originate from oleanic acid and can activate Nrf2 signaling in both cell culture and animal models, exhibiting antiinflammatory and antioxidant activities.^14^, ^15^, ^16^ CDDO‐ethyl amide (CDDO‐EA, which chemical structure is shown as Figure 1A) has better bio‐availability and can effectively penetrate the blood‐brain barrier in mice.^17^, ^18^ However, it is not known whether CDDO‐EA confers neuroprotection against ischemic injury or its impact on microglia/macrophage polarization in the ischemic context.

In this study, we aim to explore the influence of CDDO‐EA on cerebral ischemic injury and microglia/macrophage polarization using the mice model of transient focal ischemia.

## 2 MATERIALS AND METHODS

### 2.1 Experimental design

Using an in vivo model, we first evaluated the optimal dose of CDDO‐EA (1, 2 and 4 mg/kg, i.p.) to induce Nrf2 and HO‐1 protein expression and subcellular compartmentalization in MCAO mice. Thirty mice were divided into five groups (n = 6/group): sham, MCAO + vehicle, MCAO + CDDO‐EA (1 mg/kg, 2 mg/kg, or 4 mg/kg, i.p.). CDDO‐EA was delivered 30 minutes after the end of the ischemic period (ie, after reperfusion).

We then assessed neuroprotective effect and microglial polarization in ischemic animals treated with CDDO‐EA or vehicle by examining the neurological score, infarct volume, TUNEL staining, and immunofluorescence (Figure 1B). Mice were divided into three groups (n = 12/group): sham, MCAO + vehicle, MCAO + CDDO‐EA (2 mg/kg, i.p.).

Expression levels of HO‐1, CD16, and CD11b, and microglial phagocytotic activity were determined by Western blot analysis, real‐time quantitative PCR, and fluorescent microsphere uptake in LPS‐activated BV2 microglial cells.

All animals were randomly assigned to sham, MCAO + vehicle, or MCAO + CDDO‐EA groups using a lottery‐drawing box. All experiments were performed by an investigator blinded to experimental group assignments.

### 2.2 In vivo model of transient focal ischemia

All animal experiments were approved by the Ethics Committee of the Shandong First Medical University and performed in accordance with guidelines of the Use of Experimental Animals of National Institutes of Health. Male C57BL/6 mice (10‐ to 12‐week old, 22‐30 g) were purchased from Pengyue Laboratory Animal Breeding Co., Ltd. Mice were deeply anesthetized with 3% isoflurane and maintained with 1.5% isoflurane in oxygen/nitrous oxide (30%:70%) by a rodent ventilator (RWD, China), and focal ischemia was induced by intraluminal occlusion of left middle cerebral artery (MCAO) as in our previous study.^19^ Briefly, a midline cervical skin incision was made under the surgical microscope; then, the underlying muscular attachment was separated to expose the left common carotid artery (CCA), the internal carotid artery (ICA) and its pterygopalatine artery branch, and the external carotid artery (ECA). The superior thyroid and distal ECA were permanently coagulated, but the other arteries were only temporarily ligated. A 6‐0 nylon suture was inserted into the left ICA through a dissected ECA, which was advanced 9 mm to arrive the bifurcation of the anterior cerebral artery. The animals underwent MCAO for 60 minutes after which the suture was removed, allowing for reperfusion. To confirm the occurrence of MCAO, changes in local cerebral blood flow (CBF) were measured using a laser Doppler blood flow imager (MoorLDI2). Animals that died or failed to show a CBF reduction of at least 75% were excluded from further experimentation. In sham‐operated groups, mice were anesthetized, the ECA branches were dissected, and the wound was sutured. Thirty min after reperfusion, CDDO‐EA was administered i.p. to treatment groups.

### 2.3 Western blot

Western blot analysis was performed according to previous reports.^9^, ^20^ The peri‐infarct region of the cortex from the left hemisphere was harvested 48 hours after ischemia. Whole‐cell lysates were prepared using an RIPA Lysis Buffer (P0013, Beyotime). Cytoplasmic and nuclear fractions were extracted using a nuclear and cytoplasmic protein extraction kit for subcellular fractionation (P0028, Beyotime). Equal amounts protein (30 µg) was loaded and separated in 10%‐12% (v/v) SDS gel, and probed with primary antibodies recognizing Nrf2 (1:1000, ab31163, Abcam), HO‐1 (1:1000, ADI‐OSA‐110‐D, Enzo Life Science), Histone H3 (1:1000, 9715, Cell Signaling Technology), and β‐actin (1:2000, A1978, Sigma‐Aldrich) for 12 hours at 4°C. After rinses, the blots were incubated with rabbit or mouse IgG horseradish peroxidase‐linked secondary antibody (1:4000, Cell Signaling Technology) for 2 hours. Immunopositive bands were visualized by chemiluminescence substrate (34080, Thermo Fisher) in ChemiDoc™ MP imaging System (Bio‐Rad) and then analyzed with Image J software.

### 2.4 Neurological score

After MCAO mice were awake for 24 and 48 hours, neurological deficit was scored using the modified Longa method (Table 1) according to a previous report.^13^ The mice were randomly assigned to MCAO + vehicle (Vehicle) and MCAO + CDDO‐EA (CDDO‐EA) groups using a lottery‐drawing box. All of the outcome assessments were performed by investigators blinded to the group assignments.

**TABLE 1:** Longa neurological score

Score	Behavior
0	No deficits
1	Mice with difficulty in extending contra‐lateral forelimb
2	Mice with mild circling to the contra‐lateral side
3	Mice with severe circling
4	Mice with no spontaneous motor activity
5	Death

### 2.5 Assessment of cerebral infarct

2, 3, 5‐triphenyltetrazolium chloride (TTC) staining was used to assess the infarct size according to a previous study.^19^ The forebrains of mice were removed at 48 hours after MCAO under anesthesia with 3% isoflurane. The infarct volume was measured by 2% TTC (Sigma‐Aldrich) staining on 2‐mm‐thick coronal fresh brain sections. Normal brain area was stained red, while the infarct area not stained (white). The infarct volume is quantified from the stained sections by using ImageJ software.

### 2.6 Immunofluorescent and TUNEL staining

Immunofluorescent and Terminal deoxynucleotidyl transferase dUTP nick end labeling (TUNEL) staining was performed as in previous studies.^6^, ^19^ Briefly, 48 hours after sham or MCAO‐operated surgery, mice were anesthetized by 3% isoflurane and transcardially perfused with 4% paraformaldehyde/PBS buffer. Brains were dehydrated in 30% sucrose/PBS buffer. A total of 10‐μm‐thick coronal fresh brain sections were obtained using a Leica CM1950 cryostat. permeabilized with 0.5% Triton X‐100, and blocked in 5% goat serum. For immunohistochemistry, sections were incubated with primary antibodies (NeuN (1:300, MABN140, Millipore); Iba‐1 (1:300, 19741, Wako); CD16 (1:300, 553142, BD Biosciences); and CD206 (1:300, AF2535, R&D Systems) for 16 hours at 4°C, followed by secondary antibodies (488‐conjugated donkey anti‐mouse IgG or Cy3‐conjugated donkey anti‐rabbit IgG, 1:800, Jackson Immunoresearch). TUNEL staining was performed with an in situ cell death detection kit with fluorescein (11684795910, Roche) following the manufacturer's instruction. After that, sections were washed and cover slipped with antifading buffer and viewed through a fluorescent microscope (Olympus company BX51) using standardized parameters. The number of positive cells was quantified and averaged as cells per mm^2^.

### 2.7 Microglia culture, cell treatment, and phagocytosis assay

Mouse microglia BV2 cells were obtained from China Academia Sinica cell repository (Shanghai, China), and maintained in Dulbecco's modified Eagle's medium (DMEM, Gibcao) supplemented with 10% fetal bovine serum (FBS) and incubated at 37°C with 5% CO_2_ in 6 cm plates. Cells were plated 24 hours prior to stimulation at a confluency of 80%, and treated with CDDO‐EA (0, 50, 100 and 200 μg/mL, HY‐12213, MedChemExpress), LPS (100 ng/mL, L4391, Sigma‐Aldrich), LPS (100 ng/mL) + CDDO‐EA (100 μg/mL), or LPS (100 ng/mL) + tin‐protoporphyrin IX (Sn‐PPIX, a specific HO‐1 inhibitor, 10 μg/mL, HY‐101194, MedChemExpress) for 24 hours. For the phagocytosis assay, fluorescent microspheres (1:10 000, F8819, Invitrogen) were added into microglial cultures for 3 hours and detected within cell bodies using confocal microscopy (Nikon A1).

### 2.8 Real‐Time quantitative PCR

Real‐time quantitative PCR was performed as in a previous report.^6^ Total RNA (4 μg) was extracted from BV2 cells and reversed transcribed into cDNA using SuperScript III First‐Strand System (18080‐051, Invitrogen). PCR was performed on a real‐time PCR detection system (Bio‐Rad) using corresponding primers (Table 2) and SYBR Green FAST Mastermix (330603, QIAGEN). The two‐step amplification protocol consisted of denaturation for 30 s at 94°C, followed by 32 cycles of 95°C for 5 s and 57°C for 60 s. RNA quantity was calculated using the Ct method, normalized and expressed as fold change as compared to control.

**TABLE 2:** Primers for real‐time polymerase chain reaction

Gene	Primer (sense)	Primer (reverse)
M1 phenotype		
CD16	TTTGGACACCCAGATGTTTCAG	GTCTTCCTTGAGCACCTGGATC
CD11b	CCAAGACGATCTCAGCATCA	TTCTGGCTTGCTGAATCCTT
M2 phenotype		
CD206	CAAGGAAGGTTAACATTTGT	CCTTTCAGTCCTTTGCAAGC
CCL22	CTGATGCAGGTCCCTATGGT	GCAGGATTTTGAGGTCCAGA

### 2.9 Statistical analysis

Data were analyzed with GraphPad Prism 6.0 Software and expressed as mean ± SD (standard deviation). Levene test was used for assessing the homogeneity of variance. Student two‐tailed *t* test was used for the comparison of two experimental groups when the data were normally distributed. Wlicoxon rank‐sum test was used for data comparisons with non‐normal distributions. *P* value < 0.05 was considered statistically significant.

## 3 RESULTS

### 3.1 CDDO‐EA increases Nrf2 and HO‐1 in cortex after MCAO

We first wished to determine the optimal dose of CDDO‐EA in order to induce Nrf2 protein expression following MCAO. Mice were administered CDDO‐EA (0, 1, 2, and 4 mg/kg, i.p.) 30 minutes after the onset of reperfusion; then, ischemic cortices were isolated and processed for Western blot 48 hours after ischemia. Nrf2 was clearly detected in sham brain, and vehicle treatment in the MCAO group did not significantly increase the expression of Nrf2 as compared with the sham‐operated group. However, Nrf2 expression significantly increased in the CDDO‐EA‐treated MCAO groups (2 and 4 mg/kg, i.p.) compared to the vehicle‐treated MCAO group (Figure 1C and Figure S1A). No significant difference between the two higher doses of CDDO‐EA was observed (Figure 1C, middle panel). Using the 2 mg/kg dose of CDDO‐EA, we next performed subcellular fractionation to determine whether Nrf2 is enriched in the nuclear fraction in ischemic brain, which is reflective of the dissociation of Nrf2 with tethering proteins in the cytosol. Neither sham nor vehicle‐treated MCAO brain exhibited substantial levels of Nrf2 protein in the nuclear fraction, indicating that Nrf2, although present at the protein level in neural cells, does not exhibit robust nuclear localization and likely remains tethered in the cytosol. However, in animals receiving 2 mg/kg CDDO‐EA at 30 minutes following reperfusion, the detectable level of Nrf2 protein in the nuclear fraction significantly increased (Figure 1D and Figure S1B). These results indicate that treatment with CDDO‐EA following ischemic stroke can both increase total Nrf2 protein expression in brain and increase nuclear translocation of Nrf2 protein, consistent with a possible increase in transactivational activity.

HO‐1 is a transcriptional target of nuclear Nrf2 transactivational activity; thus, increased expression of HO‐1 is considered consistent with Nrf2 nuclear activity. Following MCAO in vehicle‐treated mice, HO‐1 protein expression increased significantly compared to sham controls (Figure 1C), reflecting endogenous processes are active in ischemic brain that increase HO‐1 expression 48 hours after MCAO. Administration of CDDO‐EA 30 minutes following MCAO significantly up‐regulated HO‐1 protein expression, consistent with the up‐regulation and nuclear localization of Nrf2 protein (Figure 1C). Furthermore, CDDO‐EA increased the presence of HO‐1 in the cytosol 48 hours after MCAO (Figure 1D). Together, these data indicate that CDDO‐EA stimulates Nrf2 protein expression and activation, evidenced by the increased protein expression levels of the Nrf2 target, HO‐1.

### 3.2 CDDO‐EA improves neurological score and reduces neuronal death in MCAO mice

**FIGURE 2:**
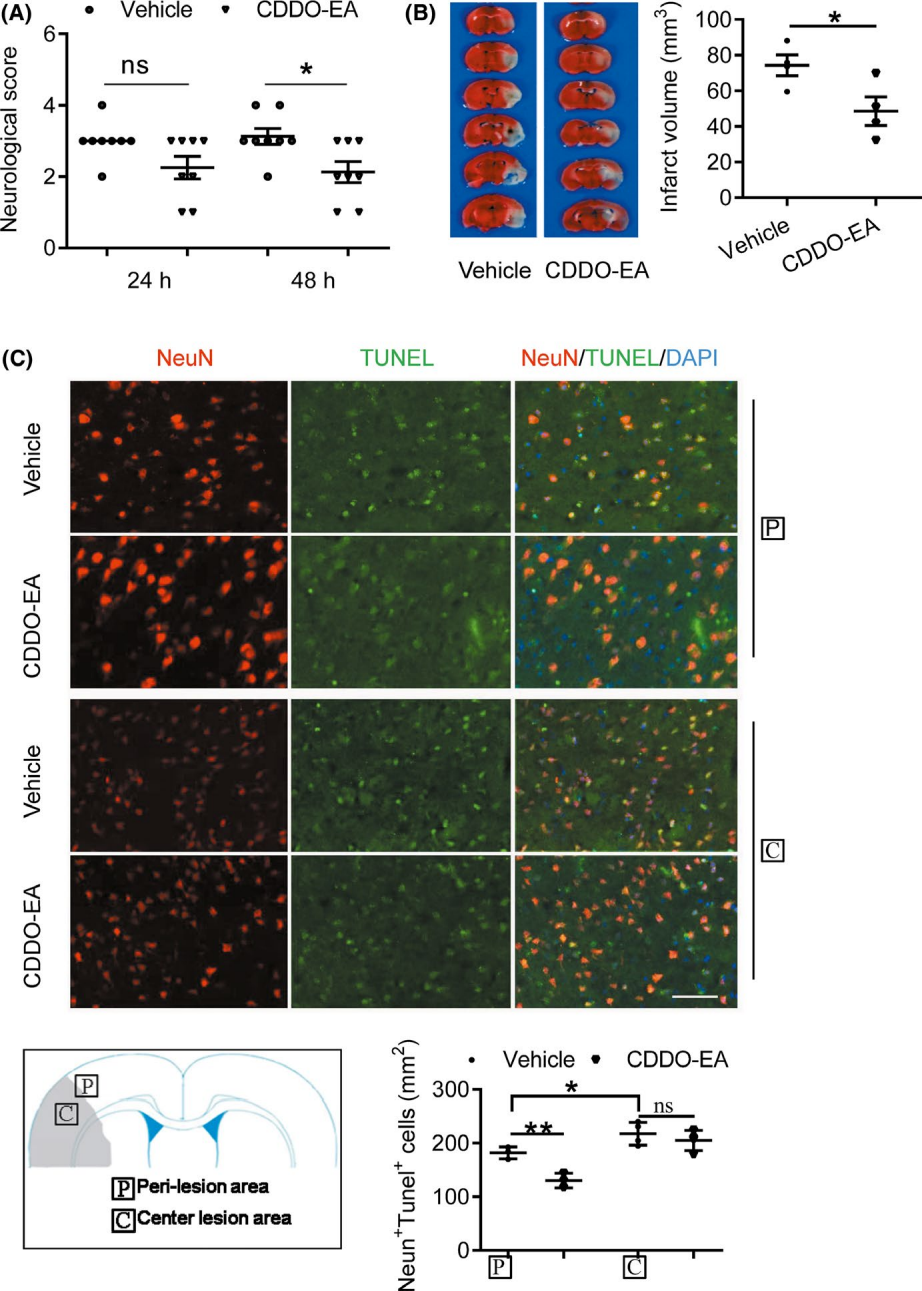
CDDO‐EA reduces neurological deficit, infarct volume, and neuronal death following ischemic injury in mice. A, Assessment of neurological deficits in mice using a modified Longa score 24 and 48 h following MCAO. B, Representative images of TTC‐stained brain coronal sections obtained 48 h following transient MCAO with vehicle or CDDO‐EA for the determination of total infarct volume. C, Coronal sections from MCAO + Vehicle and MCAO + CDDO‐EA group on 48 h after MCAO were subjected to TUNEL staining (green) and immunostaining for the NeuN (red) in center lesion area and peri‐lesion area of ischemic cortexes. Quantification was performed by counting the TUNEL^+^/NeuN^+^ neurons in the indicated region and expressed as cells per mm^2^. *Scale bars* = 20 μm. Data are mean ± SD. Data were analyzed using an unpaired t test with Welch correction (ns denotes not significant, ^*^ *P* < 0.05, ^**^ *P* < 0.01, n = 8 in A, n = 4 in B and C)

Acute neurological function was assessed in mice 24 and 48 hours after MCAO or sham surgery, using a modified Longa method. Compared to the sham‐operated group, neurological scores significantly worsened in the vehicle‐treated MCAO group (Figure 2A). Postischemic treatment with CDDO‐EA (2 mg/kg, i.p.) significantly improved neurological scores at 48 hours, but not 24 hours, following MCAO compared to the vehicle‐treated group (Figure 2A). Examination of total tissue infarct volume by TTC staining indicated that the CDDO‐EA‐treated group exhibited significantly smaller infarct volume at 48 hours following MCAO as compared to the vehicle‐treated MCAO group (Figure 2B). Furthermore, histological analyses of NeuN (red) and TUNEL (green) double staining in the penumbral region demonstrated that neuronal cell death (number of NeuN^+^/TUNEL^+^ cells) was significantly reduced in CDDO‐EA‐treated compared to vehicle‐treated MCAO mice (Figure 2C). However, the central lesion area was not significantly different in terms of the number of NeuN^+^/TUNEL^+^ cells between CDDO‐EA‐treated and vehicle‐treated groups.

### 3.3 CDDO‐EA primes microglia/macrophages toward an M2 phenotype in MCAO brain

**FIGURE 3:**
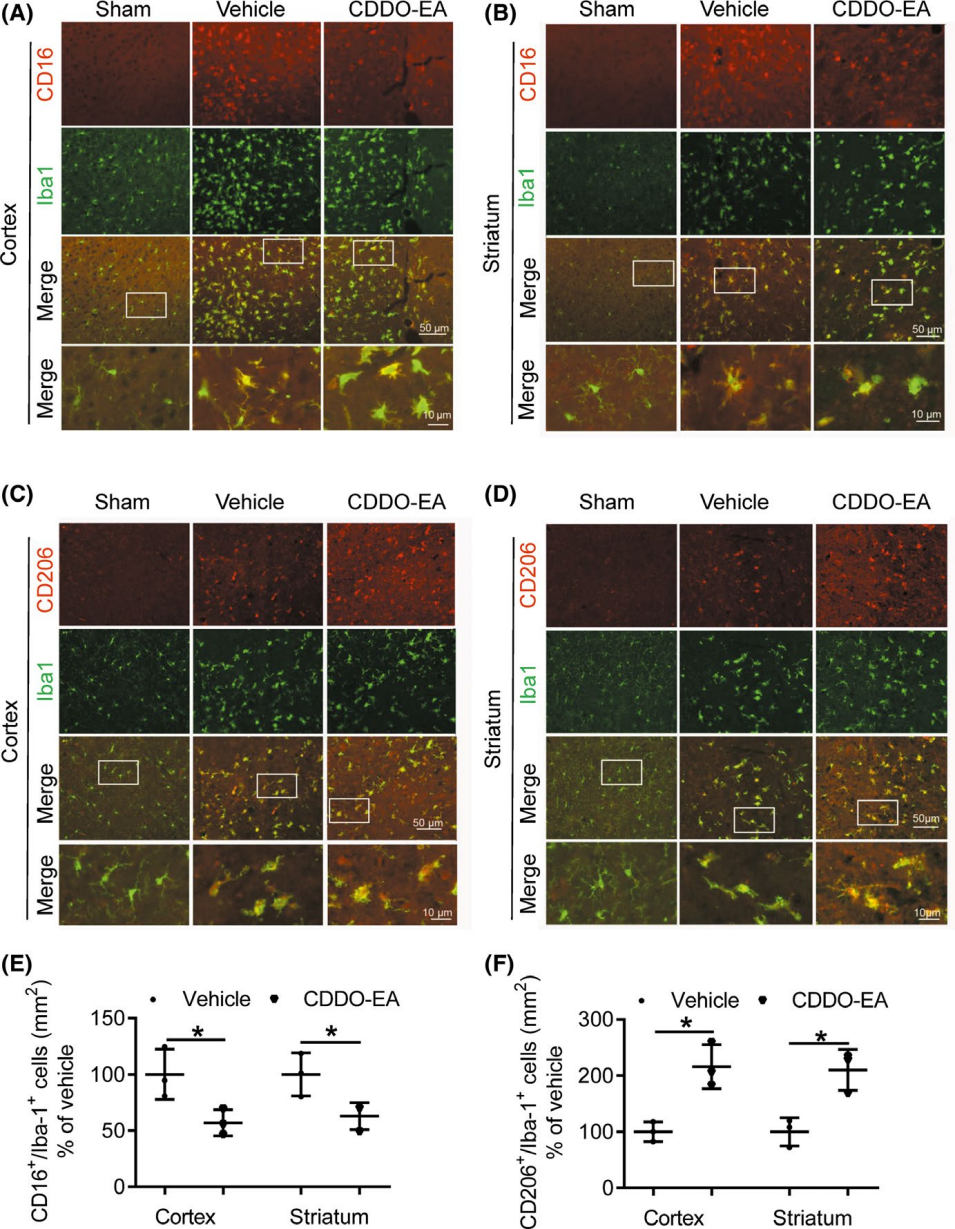
CDDO‐EA is associated with decreased M1 phenotype expression and increased M2 phenotype expression in microglia/macrophages within the ischemic cortex after MCAO. A–D, Coronal sections from MCAO + Vehicle and MCAO + CDDO‐EA cortex and striatum 48 h after MCAO were subjected to immunostaining for either CD16 or CD206 (red) and for Iba 1 (green) 48 h after MCAO. E, F, Quantification was performed by counting the CD16^+^/ or CD206^+^/Iba1^+^ microglia/macrophages in the region (per mm^2^). *Scale bars* = 50 or 10 μm. Data are mean ± SD (^*^ *P* < 0.05, n = 3)

To investigate whether CDDO‐EA impacts the phenotypic polarization of microglia/macrophages following MCAO, the representative M1 phenotype marker protein (CD16) and the M2 phenotype marker protein (CD206) were analyzed by double immunofluorescent staining with the microglia/macrophage marker (Iba1) within the inner boundary of the infarct region. MCAO led to a qualitative increase in both CD16‐ and CD206‐positive microglia/macrophages in the cortex and striatum at 48 hours as compared to sham control (Figure 3A,B). Following MCAO, the number CD16^+^/Iba1^+^ was significantly less in the cortex and striatum from CDDO‐EA‐treated mice compared to vehicle‐treated mice (Figure 3A,B,E). Furthermore, the MCAO group treated with CDDO‐EA exhibited significantly more CD206‐positive microglia/macrophages (CD206^+^/Iba1^+^) in the cortex and striatum at 48 hours as compared to vehicle‐treated MCAO group (Figure 3C,D,F). Thus, although MCAO leads to increased numbers of both M1 and M2 polarized microglia/macrophage compared to sham surgery, postischemic treatment with CDDO‐EA appears to shift the polarization of microglia/macrophage toward an M2 phenotype.

### 3.4 CDDO‐EA increases HO‐1 expression and promotes M2 polarization in LPS‐stimulated microglia

**FIGURE 4:**
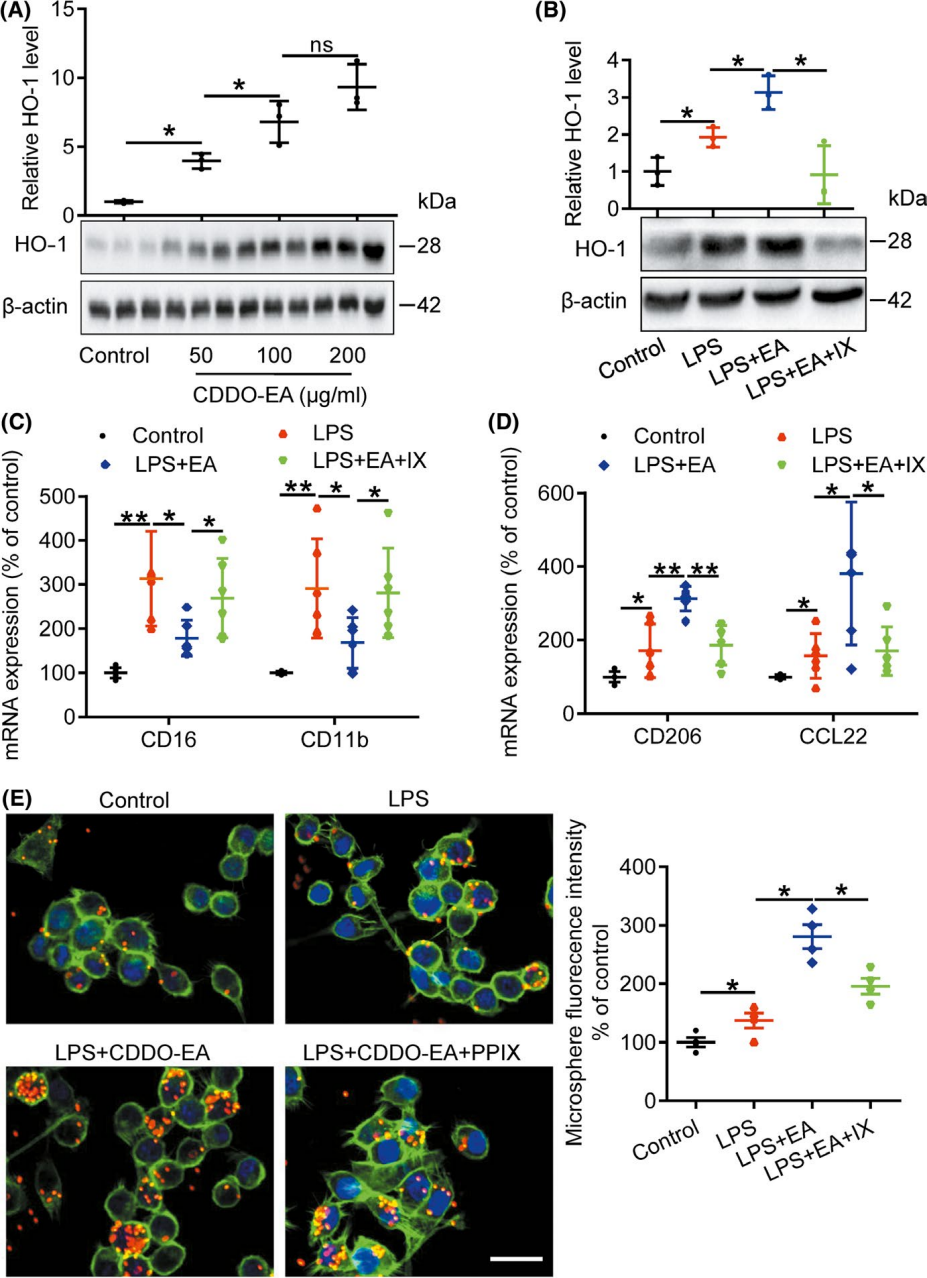
CDDO‐EA increases expression of HO‐1, CD206, and CCL22, reduces CD16 and CD11b expression, and enhances microglial phagocytosis in LPS‐activated BV2 microglial cells. A, B, Cells were treated with CDDO‐EA (0, 50, 100, or 200 μg/mL), LPS (100 ng/mL), LPS (100 ng/mL) + CDDO‐EA (100 μg/mL), or LPS (100 ng/mL) + PPIX (10 μg/mL) for 24 h, lysates collected, and homogenates were immunoblotted with anti‐HO‐1 and anti‐β‐actin. C, D, BV2 cells were treated with LPS (100 ng/mL), LPS (100 ng/mL) + CDDO‐EA (100 μg/mL), or LPS + CDDO‐EA + PPIX (10 μg/mL) for 24 h, and the level of CD16, CD11b, CD206, and CCL22 mRNA were detected with real‐time quantitative PCR. E, BV2 cells were treated with LPS (100 ng/mL), LPS (100 ng/mL) + CDDO‐EA (100 μg/mL), or LPS + CDDO‐ EA + PPIX (10 μg/mL) for 21 h, fluorescent microspheres were added into the medium for 3 h, and then, the cells were stained with phalloidin to visualize F‐actin. The left panel is representative images of intra‐microglia fluorescence 3 h after fluorescent microspheres uptake. *Scale bars* = 50 μm (ns denotes not significant, ^*^ *P* < 0.05, ^**^ *P* < 0.01, n = 3‐6)

The above in vivo results cannot determine if CDDO‐EA has a direct effect on microglia or if CDDO‐EA alters the general environment of the ischemic brain and thus elicits an indirect response from microglia/macrophage. Using cultures of BV2 microglial cells, we first determined that CDDO‐EA (0, 50, 100, or 200 μg/mL) and the inflammatory stimulus lipopolysaccharide (LPS, 100 ng/mL) were not cytotoxic to BV2 microglial cells (data not shown). We then investigated the effect of CDDO‐EA on HO‐1 expression to determine whether the increase in Nrf2‐related activity observed in vivo could be recapitulated in cultured BV2 microglial cells. Treatment of BV2 cells with CDDO‐EA (50‐200 μg/mL) alone increased the protein expression of HO‐1 in BV2 microglia in a dose‐dependent manner (Figure 4A and Figure S1C). We next sought to determine the effects of CDDO‐EA on BV2 microglia in the context of an inflammatory stimulus. Similar to the increase of HO‐1 in ischemic brain, stimulation with LPS alone led to increased HO‐1 protein expression compared to control cultures. Cotreatment with CDDO‐EA significantly increased in HO‐1 expression compared to LPS‐treated cultures (Figure 4B and Figure S1D); the expression of increased HO‐1 induced either by LPS or by combination of LPS and CDDO‐EA was suppressed by addition of the HO‐1 enzymatic inhibition Sn‐PPIX. Similar to the in vivo data presented above, CDDO‐EA, a Nrf2 activator, stimulates the expression of the Nrf2 target gene HO‐1 in cultured microglia.

As an endotoxin, LPS induces microglia toward an inflammatory state. Consistently, we found that mRNA expression of M1 markers (CD16 and CD11b) was significantly increased in LPS‐treated BV2 microglial cells (Figure 4C), as detected by real‐time quantitative PCR. Treatment with CDDO‐EA effectively reduced LPS‐stimulated mRNA expression of CD16, CD11b, and iNOS (Figure 4C and Figure S2A). In contrast, CDDO‐EA significantly increased expression of M2 markers (CD206, CCL22, and IL‐10) in LPS‐treated BV2 microglial cells (Figure 4D and Figure S2B). Cotreatment with the HO‐1 inhibitor Sn‐PPIX with LPS and CDDO‐EA diminished the effects of CDDO‐EA on BV2 polarization (Figure 4C,D). Moreover, a fluorescent microsphere engulfment assay demonstrated that CDDO‐EA treatment significantly enhanced the phagocytic capacity of LPS‐treated BV2 microglial cells (Figure 4E); however, the effect of CDDO‐EA was significantly decreased with cotreatment of Sn‐PPIX (Figure 4E). Together, these data indicate that CDDO‐EA directly impacts microglial cultures exposed to an inflammatory stimulus by increasing HO‐1 expression, promoting polarization toward an M2 phenotype, and enhancing phagocytic activity, all dependent at least in part on HO‐1 activity.

## 4 DISCUSSION

The effects of postischemic administration of the Nrf2 activator CDDO‐EA on microglial activation have not been explored. We detail here the novel finding that intraperitoneally administered CDDO‐EA reduced cerebral infarct volume and neural cell apoptosis after transient MCAO, significantly increased expression and subcellular localization of Nrf2 and HO‐1, and promoted microglia/macrophage polarization toward M2 phenotype after transient MCAO. These observations were recapitulated in a culture model using LPS‐stimulated microglia BV2 cells. These findings suggest that the Nrf2 activator CDDO‐EA provides neuroprotection after MCAO in mice and may function directly on microglia in promoting an antiinflammatory environment.

Under physiological conditions, the transcription factor Nrf2 is inactive and binds to Kelch‐like erythroid cell‐derived protein with CNC homology‐associated protein 1 (Keap1) in cytoplasm.^21^, ^22^ Upon oxidative stress, Nrf2 is activated and released from Keap1, then translocates to nucleus and binds to ARE, and activates expression of its downstream genes including phase II detoxification enzymes, thioredoxin, and HO‐1, which elicits cyto‐protective effects against oxidative stress.^22^, ^23^ CDDO originates from oleanolic acid that has been used to treat hepatitis in China^24^ and exhibits strong anticancer, antiinflammatory, and antioxidant activities.^18^ CDDO derivatives, such as CDDO‐Im (CDDO imidazolide), CDDO‐Me (CDDO‐methyl ester), and CDDO‐EA, are potent activators of Nrf2 signaling.^18^ Numerous studies have reported neuroprotective effects of CDDO‐EA in Huntington disease^25^ and amyotrophic lateral sclerosis^17^ animal models. We now add to this literature that CDDO‐EA alleviates ischemic injury in the transient MCAO mice model. The ability of CDDO‐EA to activate Nrf2 and induce its downstream antioxidant target genes is a well‐established mode of action. Similarly, our results demonstrate that postischemic CDDO‐EA treatment is associated with increased Nrf2 expression and nuclear translocation, and up‐regulation of the Nrf2 target gene HO‐1 in the transient MCAO mice model.

Cerebral ischemia causes glial and neuronal cell death, leading to local inflammation in brain parenchyma and microvasculature characterized by production of proinflammatory factors and rapid activation of resident microglia as well as peripheral leukocyte infiltration into the ischemic lesion.^26^ Microglia‐mediated neuroinflammation plays an important role in the pathogenesis and progression of ischemia/reperfusion injury. Activated microglia/macrophages have been characterized by the proinflammatory M1 phenotype, which expresses differentiation markers CD86 and CD16/32, or the antiinflammatory M2 phenotype, which expresses arginase‐1 (Arg1) and CD206.^27^ Several reports have indicated that polarization of microglia/macrophages affect neuroinflammation after ischemic brain injury. Reduction of endothelial microRNA‐126 expression evokes microglial activation and induces neuroinflammation in a multiple microinfarction model.^28^ Disrupted CX3CR1‐CCR2‐dependent signaling alters the phenotype of microglia/macrophages and contributes to neuroinflammation in a mouse model of childhood stroke.^29^ Transient selective brain cooling promotes polarization of microglia/macrophages to antiinflammatory phenotypes in a tMCAO model.^30^ In our study, pharmacological stimulation of Nrf2 activation promotes polarization of microglia/macrophages toward an antiinflammatory M2 phenotype after ischemic injury. Similar to other reports,^6^, ^31^ our results suggest that cerebral ischemia causes an significant increase in the number of CD16‐positive microglia/macrophages, while CDDO‐EA reduces the number of these M1 phenotype cells. The M2 microglial/macrophage phenotype promotes neurogenesis, angiogenesis, axonal remodeling, and remyelination in several models of acute CNS injury, including spinal cord injury, traumatic brain injury, and stroke.^5^, ^32^ However, all of these models are complicated by the diverse cell types that may be directly or indirectly affected by CDDO‐EA. Previous studies have demonstrated that activation of Nrf2 shifts microglia/macrophages polarization toward an antiinflammatory M2 phenotype.^7^, ^8^, ^33^ We present here that CDDO‐EA leads to an increase in the number of CD206‐positive microglia/macrophages in brain, but further extended the in vivo findings to demonstrate a direct effect of CDDO‐EA on microglial BV2 cells in culture. Our results showed that CDDO‐EA cotreatment with the endotoxin LPS up‐regulated the expression of the Nrf2 target protein HO‐1 and promoted a phenotypic change in BV2 cells, including the down‐regulation of mRNAs of M1 phenotype markers and enhancement of the mRNAs of M2 phenotype markers. These data are consistent with several studies reporting that activation of Nrf2 and its downstream HO‐1 can suppress LPS‐mediated neuroinflammation both in vivo and in vitro.^34^, ^35^, ^36^ After ischemic injury, activation of macrophage/microglia plays a major role in phagocytic clearance and resolution of neuroinflammation. It is worth noting that macrophage/microglia display different molecular patterns and phenotypes depending on aging and sex,^37^, ^38^, ^39^ and indeed, age and sex may influence the protective effect and efficacy of CDDO‐EA. Further studies are needed to exclude these possibilities in middle‐aged and elderly female animals.

In summary, this study describes a therapeutic effect of CDDO‐EA in a transient MCAO model of ischemic stroke, consistent with decreasing neuronal apoptosis and promoting microglia/macrophages polarization toward an antiinflammatory phenotype. Further studies are warranted to explore the effect of CDDO‐EA on long‐term functional recovery after stroke.

## CONFLICT OF INTEREST

The author(s) declared no potential conflicts of interest for the research, authorship, and publication of this article.

## Supporting information

Fig S1

Fig S2

## Data Availability

Data sharing not applicable—no new data generated.
