# Supplementary figures and images for: The novel Nrf2 activator CDDO‐EA attenuates cerebral ischemic injury by promoting microglia/macrophage polarization toward M2 phenotype in mice
Source: CNS Neurosci Ther. 2020 Dec 6;27(1):82–91. doi: 10.1111/cns.13496 (PMC7804925; doi:10.1111/cns.13496)

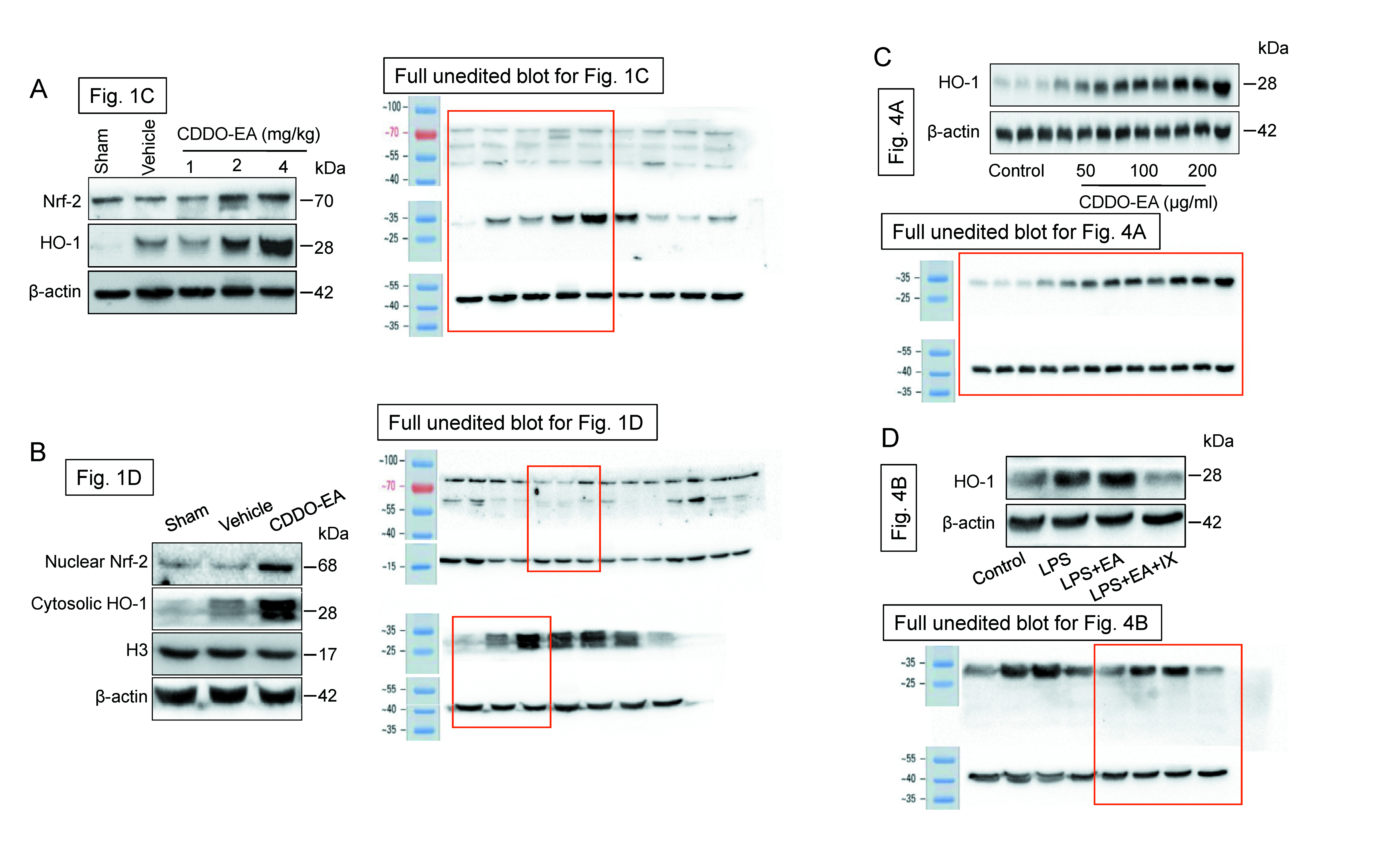

Supplement: Supplementary file 1 — Fig S1 [file CNS-27-82-s001.tif]

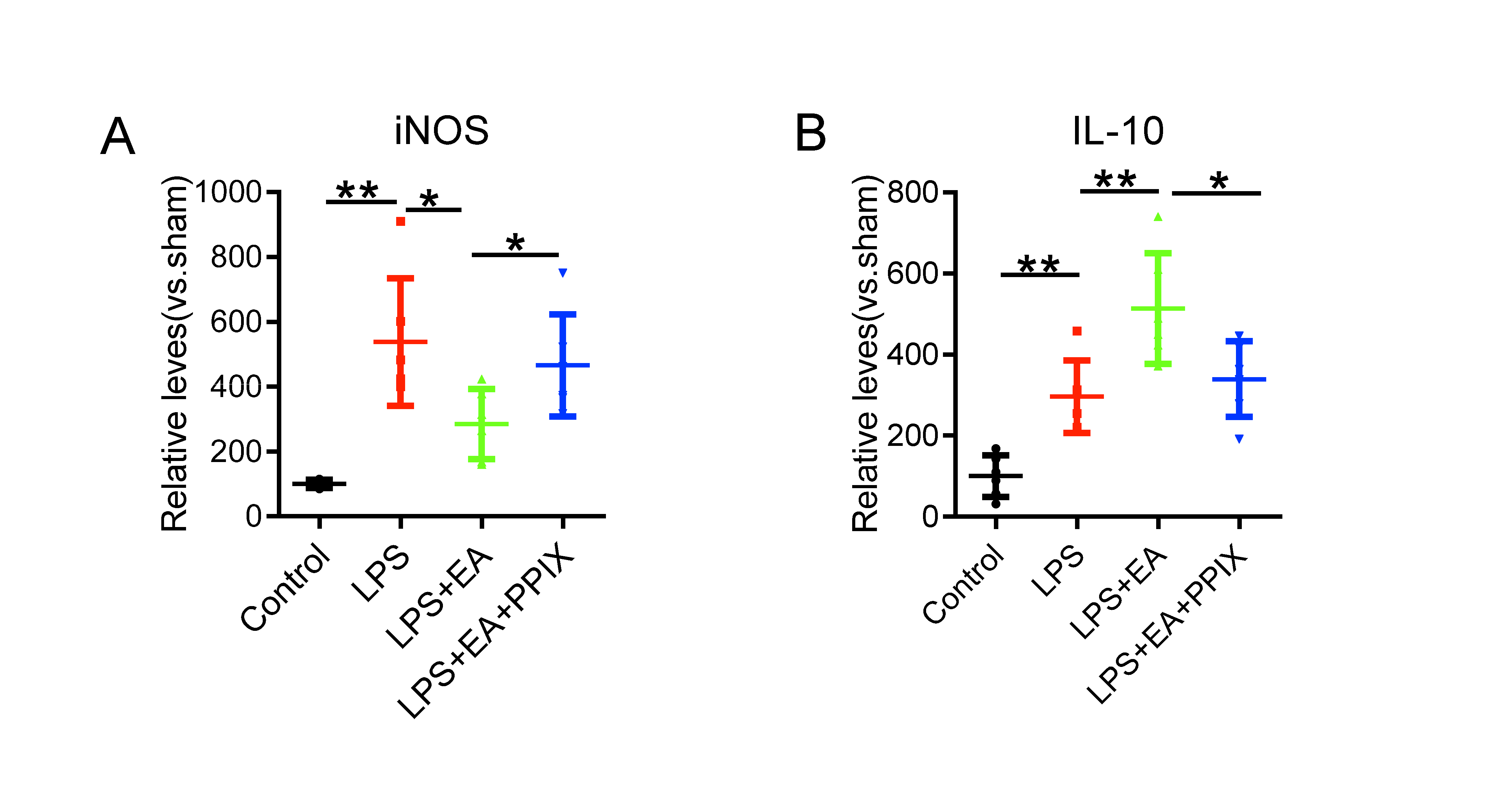

Supplement: Supplementary file 2 — Fig S2 [file CNS-27-82-s002.tif]
